# Determination of Lead Elemental Concentration and Isotopic Ratios in Coal Ash and Coal Fly Ash Reference Materials Using Isotope Dilution Thermal Ionization Mass Spectrometry

**DOI:** 10.3390/ijerph16234772

**Published:** 2019-11-28

**Authors:** Chaofeng Li, Huiqian Wu, Xuance Wang, Zhuyin Chu, Youlian Li, Jinghui Guo

**Affiliations:** 1 State Key Laboratory of Lithospheric Evolution, Institute of Geology and Geophysics, Chinese Academy of Sciences, Beijing 100029, China; wuhuiqian17@mails.ucas.edu.cn (H.W.); zhychu@mail.iggcas.ac.cn (Z.C.); lyl@mail.iggcas.ac.cn (Y.L.); jhguo@mail.iggcas.ac.cn (J.G.); 2 Innovation Academy for Earth Science, Chinese Academy of Sciences, Beijing 100029, China; 3 University of Chinese Academy of Sciences, Beijing 100049, China; 4 Research Centre for Earth System Science, Yunnan Key Laboratory of Earth System Science, Yunnan University, Kunming 650500, China; xuancewang@mail.iggcas.ac.cn; 5 School of Earth and Environmental Sciences, The University of Queensland, Brisbane, QLD 4072, Australia

**Keywords:** Pb isotope, coal fly ash, coal ash, ^204^Pb–^207^Pb double spike, TIMS

## Abstract

The rapid expansion of coal-fired power plants around the world has produced a huge volume of toxic elements associated with combustion residues such as coal fly ash (CFA) and coal ash (CA), which pose great threats to the global environment. It is therefore crucial for environmental science to monitor the migration and emission pathway of toxic elements such as CFA and CA. Lead isotopes have proved to be powerful tracers capable of dealing with this issue. Unfortunately, up to now, few high precision lead isotope data of CFA and CA certified reference materials (CRMs) determined by using the double spike technique have been reported. Hence, to facilitate the application of lead isotopes in environmental science, it is indispensable and urgent to determine a suite of high precision Pb isotope ratios and Pb elemental contents for CFA and CA CRMs. Here, we measured lead isotope ratios from four CFA and CA CRMs using thermal ionization mass spectrometry (TIMS) combined with the ^204^Pb–^207^Pb double spike method. Lead isotope ratios values of CRMs (GBW11124, GBW08401, GBW11125d, and JCFA-1) covered wide variation ranges from 17.993 to 19.228 for ^206^Pb/^204^Pb, from 15.513 to 15.675 for ^207^Pb/^204^Pb, and from 38.184 to 39.067 for ^208^Pb/^204^Pb. Lead isotope ratios of these CRMs, except for GBW11124, show good external reproducibility (2 RSD, n = 8), which is better than 0.05% for ^206^Pb/^204^Pb and ^207^Pb/^204^Pb, 0.07% for ^208^Pb/^204^Pb, 0.04% for ^206^Pb/^207^Pb, and 0.05% for ^208^Pb/^206^Pb. The Pb concentrations of these CRMs were determined using ^207^Pb single spike method. The reproducibility (1 RSD, n = 4) of Pb elemental content was <0.60%. This indicates the distribution of Pb elements in these CRMs is homogeneous. With the exception of GBW11124, the suite of CRMs can be used for determining CFA and CA matrix composition for quality control of Pb isotope analyses.

## 1. Introduction

Accumulation of toxic metals, such as lead (Pb), chromium (Cr), and cadmium (Cd), in the human body from rapid industrialization and urbanization can lead to a range of health problems and increase the risk of cancer once the safe dose levels are exceeded [1]. Especially for lead, often known as a “chemical time bomb” due to its known toxicity, lead contamination can cause adverse effects on nervous, hemopoietic, cardiovascular, and endocrine systems in the human body [2]. In ecosystems, lead is one of the most common anthropogenic contaminants, which is primarily generated by combustion of coal, vehicle emissions, and smelting of nonferrous metals [3]. As one of the classical fossil fuels in industrialization and urbanization history, coal has been the foundation of energy generation since the industrial revolution in the late 18th century. According to EIA’s International Energy Outlook 2008 [4], fossil fuels currently account for 86% of the primary energy demand (36% oil, 27% coal, and 23% natural gas), whereas renewable sources (solar, wind, geothermal, biomass, and hydroelectricity) only account for about 8%, and nuclear power for about 6%. Clearly, thus far, coal is still the primary fuel resource for global electricity production in many countries around the world [4,5,6]. In 2011, coal-fired generation accounted for 29.9% of the world’s electricity supply, and its share is anticipated to be 46% by 2030 [7]. It is estimated that coal-fired power facilities around the world generate 780 million tons of CCW (coal combustion waste) each year [6]. The most common forms of CCW include both coal ash (CA) and coal fly ash (CFA) that are industrial byproducts and derived from coal combustion in thermal power plants.

As the world’s largest producer and consumer of coal [3,7], China accounted for 50.2% [7] of the world coal consumption in 2012 and therefore is seriously contributing to atmospheric Pb pollution due to the large emission of Pb (~46,300 tons during the period of 1990–2009) from coal combustion [8]. As one of the most hazardous elements in coal, Pb can be released into the environment during coal mining, processing, and utilization. During coal combustion or pyrolysis, Pb is partly emitted into the atmosphere and partly partitioned into solid residues as CCW. The accumulation of large amounts of ash from coal combustion for electric power plant generation may cause serious contamination of Pb and other heavy metals [9,10,11,12,13,14,15,16] (such as As, Hg, Ni, Cd, and Cr) in groundwater, soil, and air, thus becoming a major environmental concern in China [2,3,8]. Hence, identification and tracing of the source of Pb contamination [2,3,8], the timing of its release, and its pollutant transport pathway and distribution into the environment are crucial issues in investigation of CA and CFA pollution.

In contrast to the other isotopic indicators, such as Li [17], Hg [18], B [19], Sr [19,20,21,22], Nd [22], etc., there is no doubt that Pb isotope is a more sensitive indicator in directly tracing Pb contamination transport pathways. There are four naturally occurring stable Pb isotopes (^204^Pb, ^206^Pb, ^207^Pb, and ^208^Pb). Their abundance varies because of different decay pathways from ^238^U, ^235^U, and ^232^Th to ^206^Pb, ^207^Pb, and ^208^Pb, respectively. Different types of rock, ore deposits, and anthropogenic sources have their own distinct Pb isotopic fingerprint. Due to their stability during physical and chemical processes, the isotopic composition of Pb are not affected by industrial or environmental processing, and retain their original characteristic ratio from their source [3,4,5,6,7,8,9,10,11,12,13,14,15,16,17,18,19,20,21,22,23]. Therefore, lead isotopic ratios can be used as an ideal “fingerprint” to directly identify lead sources and investigate lead pollution pathways in various environmental media, such as soil, sediments, atmosphere particles, coal, coal ash, and coal fly ash [3,4,5,6,7,8,9,10,11,12,13,14,15,16,17,18,19,20,21,22,23].

Certified reference materials (CRMs) play an important role in isotope ratio and elemental content analysis because CRMs are the prerequisite to examine the quality of data for unknown analysis objects [22,24,25]. In previous studies, analysts have reported many trace element data for coal, coal fly ash, and coal ash CRMs [26,27,28]. However, few high precision Pb elemental contents and the isotope ratios of CA and CFA CRMs have been reported. To monitor lead isotope data of CA and CFA samples in previous studies, silicate CRMs were employed to evaluate the quality for actual CA and CFA samples. This is an eclectic analytical scheme because the matrix composition of CA and CFA is different from silicate CRMs. CA and CFA samples are composed of amorphous inorganic components, minerals (e.g., silicates, oxides, hydroxides mainly of iron, sulfates, carbonates, phosphates, and sulfides), and organic constituents or unburnt coal [15]. To obtain accurate elemental contents or isotope ratios through bulk analysis, the best method is to employ the same type of CRMs to verify the actual analytical objects. Therefore, it is crucial to report the Pb isotope composition and Pb elemental content of CFA and CA CRMs.

This study is the first to report high precision Pb isotope ratio data for CA and CFA CRMs using the ^204^Pb–^207^Pb double spike method by thermal ionization mass spectrometry (TIMS). We believe that, by integrating Pb isotope data of the suite of CRMs from this study with our previously reported Sr and Nd isotope data [22], researchers can conduct more precise investigations when tracing lead emissions resulting from anthropogenic activities and also investigate the temporal and spatial variations of lead emissions in China. This will play a key role in estimating the lead emissions from different sources, and in identifying the sectors that should control lead emissions.

## 2. Experimental

### 2.1. Reagents and Materials

The analytical grade acids (8 M HBr, 29 M HF, 14 M HNO_3_, and 12 M HCl) used in this study were obtained from the China National Pharmaceutical Group Corporation and further purified by sub-boiling distillation using a DST-1000 PFA apparatus (Savillex Corporation, Eden Prarie, MN, USA). To reduce the chemical blanks of lead, ultra-pure water generated by a Milli-Q Element system was used in this work. The Bio-Rad AG1-X8 anion resin was used to separate Pb with high purity from CA and CFA matrix. A solution of 10 ppm Pb of NIST SRM981 was gravimetrically prepared to monitor the status of Triton Plus thermal-ionization mass spectrometer (TIMS). The isotopically enriched tracers included ^207^Pb (92.813%) and ^204^Pb (82.571%) were obtained from Oak Ridge National Laboratory USA. Single-element powders of these isotope tracers were weighed (to 0.01 mg precision) and dissolved by heating in 6 M HCl. Portions of these isotope tracer solutions were mixed appropriately to obtain the mixed ^204^Pb–^207^Pb tracers. The ^204^Pb–^207^Pb double spike solution was diluted to 1.011 ppm and calibrated using NIST SRM–981. The ^204^Pb–^207^Pb double spike was employed to measure the lead isotope ratios of CRMs. The ^207^Pb isotope tracer was diluted to 0.501 ppm and employed to measure the Pb elemental content of CRMs. The ^207^Pb single spike was calibrated using reverse isotope dilution against the standard solution of 10 ppm Pb of NIST SRM981. Table 1 lists the Pb isotopic compositions of the ^207^Pb single spike and the ^204^Pb–^207^Pb double spike.

### 2.2. Coal Fly Ash and Coal Ash Certificate Reference Materials

In this study, four CA and CFA CRMs were measured. These CRMs included GBW11125d (coal ash), GBW11124 (coal ash), and GBW08401 (coal fly ash) from the Chinese National Research Centre for Certified Reference Materials (NRCCRM), and JCFA–1(coal fly ash) from the Geological Survey of Japan (GSJ). All CRMs are commercially available. Chinese CRMs can be obtained from Booming Technology Limited Inc. (http://www.boomingtec.com; email: tengqing2004@sina.com) and the Japanese CRM can be purchased from GSJ. (https://gbank.gsj.jp/geostandards/welcome.html; email: t-okai@aist.go.jp). Two basalt rock CRMs (JB-3 and BCR-2) from the GSJ and the USGS were employed to verify Pb isotope composition and Pb elemental content during sample digestion, chemical separation and TIMS measurement.

### 2.3. Sample Digestion

To avoid the potential contamination of CFA and CA samples during sample digestion and chemical separation, all sample treatments were carried out in a class 1000 clean laboratory. All samples were digested twice individually for Pb elemental content measurement and Pb isotope composition measurement, respectively. The one adding a ^207^Pb single spike was for obtaining the content of Pb. The other one without adding any spike was used to obtain high precision Pb isotope ratios. The unspiked sample after purification was divided into two aliquots, one measured directly and the other one was mixed with the ^204^Pb–^207^Pb double spike and measured [29,30].

To determine the Pb elemental content and Pb isotope ratios, 100–105 mg of powdered rock sample was weighed and added to a 7 mL round bottom Savillex Teflon screw-top capsule, and then an acid mixture of 3.0 mL of 29 M HF +0.3 mL of 14 M HNO_3_ was added and dissolved on a hotplate at 180 °C for four days. After cooling, the capsule was opened and evaporated to dryness at ca. 120 °C. Then, the sample was re-dissolved once more in 1.0 mL of 6 M HCl and reheated to 180 °C for 3 h to destroy fluoride complexes. Finally, the vials were opened and the resulting sample solution was evaporated to dryness and then re-dissolved with 1.0 mL of 0.7 M HBr on a hotplate at 120 °C.

### 2.4. Pb Purification Using Column Chemistry and Procedural Blank

Table 2 lists the detailed separation procedure for Pb. The AG1-X8 anion resin column is used to obtain the Pb fraction with high purity from the CA and CFA matrix solution. As shown in Table 2, the resulting sample solutions obtained from the previous step were loaded into a small polyethene plastic column that is 4 cm long with a 2 mm i.d. and 2 mL reservoir, packed with 0.25 mL of Bio-Rad AG1–X8 anion resin (200–400 mesh). The column was pre-cleaned with 4.0 mL 6M HCl, 5.0 mL Mill-Q H_2_O, and 1.0 mL 0.7M HBr. Considering our small column capacity, only half of the sample solution (0.50 mL of 0.7M HBr), was loaded into the AG1 column. When the sample solution was loaded into the AG1 anion resin exchange column in dilute hydrobromic acid, most matrix cations (Al, Fe, Na, K, Ti, Mg, Ca, Mn, Sr, Ba, etc.) passed through the column without interacting with the anion exchanger, whereas the Pb were strongly retained on the AG1 resin. The resin column was then washed with a further 4.2 mL of 0.7 M HBr to remove the remaining unwanted matrix elements absorbed by AG1 anion resin. Finally, the Pb fraction was eluted using 1.2 mL of 6 M HCl. The separation procedure of Pb using AG1 anion resin column in this study was similar to the previous method [31,32,33,34].

The recovery yield of Pb was higher than 82%. The whole procedure blank was lower than 140 pg for Pb. Hence, the ratio of CFA and CA sample over blank was never lower than 13,000 even for GBW08401 with relatively low content of Pb. This shows that the blanks during sample digestion and column chemistry were negligible relative to the loading size containing an aliquot of 50.0–52.5 mg CA and CFA.

### 2.5. Thermal Ionization Mass Spectrometry Measurement

Lead isotopic measurements were conducted on a Thermo Triton Plus thermal ionization mass spectrometer (TIMS) equipped with nine Faraday collectors at the Institute of Geology and Geophysics, Chinese Academy of Sciences (IGGCAS). Faraday cup collectors with 10^11^ Ω resistor were employed for Pb isotopic determinations. Re ribbon (0.035 mm thick, 0.77 mm wide, and 99.98% pure, H. Cross Company) was used as the filament material.

As for Pb elemental content measurements, all Pb samples were dissolved in 1 μL of 2.5 M HCl and loaded onto a single Re filament along with 1 μL of 0.20 M H_3_PO_4_ and 1 μL of silicate gel solution contained 5 μg SiO_2_ [29,30,31] to enhance Pb emission. The loading blank in this study was about 0.73 ± 0.09 pg (n = 3) from the mixture of 1 μL of silicate gel +1 μL of 0.20M H_3_PO_4_. As for Pb isotopic ratio measurements, the loading and measurement methods were the same as in the above description. The difference was that each chemically purified Pb sample was split into two batches in a ratio of 1:2. One batch containing ~33% was measured without adding ^204^Pb–^207^Pb double spike. The other batch containing ~67% was measured adding a suitable amount of ^204^Pb–^207^Pb double spike [29,30]. All data were acquired when the signal intensities of ^208^Pb was higher than 4.5 × ^−11^A. Each analysis included 100 ratios and was conducted in static multi-collection mode with the collector array shown in Table 3. The tuning speed of the filament current was 400 mA/min. The ionization temperature of Pb was about 1220–1300 °C.

Considerable literature now exists on the use of the double spike methods [29,30,31,34] since the early 1960s [35] and hence this is not repeated here. The final Pb isotopic data reduction was conducted off-line using the method reported by Woodhead et al. [31]. After mass fractionation correction using the ^204^Pb–^207^Pb double spike method, typical internal precision (2 RSE) was better than 0.004% for ^206^Pb/^204^Pb, ^207^Pb/^204^Pb, and ^208^Pb/^204^Pb, and 0.002% for ^206^Pb/^207^Pb and ^208^Pb/^206^Pb for most samples. NIST SRM 981 was repeatedly measured during all runs. Replicated analyses (n = 8) of the Pb reference material NIST SRM 981 with a 20 ng sample size using the ^204^Pb–^207^Pb double spike technique yielded good external reproducibility of <0.032% (2 RSD) for Pb isotope ratios, e.g., ^206^Pb/^204^Pb = 16.933 ± 0.003, ^207^Pb/^204^Pb = 15.485 ± 0.003, ^208^Pb/^204^Pb = 36.681 ± 0.012, and ^207^Pb/^206^Pb = 0.9145 ± 0.0002, showing good agreement with published data within analytical error [29,30,31,32,33,34,36,37,38].

## 3. Results and Discussion

### 3.1. Pb Elemental Concentration Results

The isotope dilution mass spectrometry (IDMS) method has been recognized as the most important technique for accurate measurements of elemental content at the highest metrological level for more than 50 years. The IDMS method only requires the high precision of isotopic ratios after sample and spike weighing and their complete mixing [39]. In contrast to other analytical techniques, the merit of IDMS is that even part loss of analyte during sample treatment does not affect the accuracy of the analytical result. For this reason, IDMS is considered a benchmark technique of elemental content analysis for CRMs [22,24,25,28,39]. It is especially true for ID combined with TIMS (ID-TIMS method) due to its high precision and accuracy. As to the principle behind single isotope dilution mass spectrometry, detailed descriptions were reported by Krata et al. [24], Vassileva et al. [25], Vogl [39], and Berglund [40]. Our calculation method was similar to these methods. Lead concentration in CA and CFA CRMs can be calculated from Equation (1):

C_x_ = C_y_ × [M_y_/M_x_] × [R_y_–R_xy_]/[R_xy_–R_x_] × [ΣR_ix_]/[ΣR_iy_] (1)

where C_x_ is the Pb elemental concentration of the sample (normally expressed as μg/g); C_y_ is the Pb elemental concentration in the spike solution; M_x_ and M_y_ are the weights of the sample and spike, respectively; R_x_ and R_y_ are the ^207^Pb/^206^Pb isotope ratio of the sample and spike, respectively; R_xy_ is the ^207^Pb/^206^Pb isotope ratio of the sample-spike mixture; and ΣR_ix_ and ΣR_iy_ are the sums of ratios for all Pb isotopes relative to the reference isotope (^206^Pb) for sample and spike, respectively.

For the ID-TIMS method, the key factor is to obtain highly reproducible and accurate Pb isotope ratios. In this case, we chose ^206^Pb as the denominator because the mass fractionation of ^207^Pb/^206^Pb is small and the reproducibility of ^207^Pb/^206^Pb is excellent. For example, the ^207^Pb/^206^Pb value for the NIST 981 standard with 20 ng sample size is 0.91384 ± 0.00038 (n = 36, 2 SD) even without any mass fractionation correction in the last year. Clearly, the reproducibility of ^207^Pb/^206^Pb is stable and better than 0.042% (2 RSD) even without any mass fractionation correction, based on long-term observations in our laboratory. We adopted the linear law to correct mass fractionation for Pb elemental content measurements. In this study, the mass fractionation factor for Pb is 0.0923%/amu, which was calculated and normalized to the recommended value (^207^Pb/^206^Pb = 0.9147) reported by Woodhead et al. [31].

**Figure 1:**
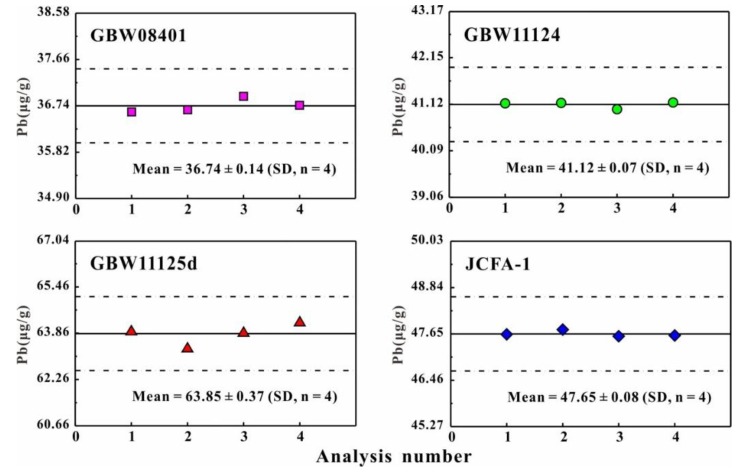
Pb elemental contents in four CFA and CA reference materials. The dashed line area defines the error of ±2% (1 RSD) of Pb elemental contents.

As shown in Table 4, the Pb contents of JB-3 and BCR-2 utilizing the ^207^Pb single spike method combined with TIMS analysis show a good agreement with previously published data [32,41,42,43,44,45] within uncertainties. This demonstrates our analytical method based on the single ^207^Pb spike in conjunction with TIMS to measure Pb elemental content is reliable and accurate. As shown in Table 4 and Figure 1, the concentration of Pb in CA and CFA reference materials was found to be 36.74 ± 0.14 μg/g for GBW08401, 41.12 ± 0.07 μg/g for GBW11124, 63.85 ± 0.37 μg/g for GBW11125d, and 47.65 ± 0.08 μg/g for JCFA-1. Lead contents for these CRMs show a relatively wide range of 36.74–63.85 μg/g. The reproducibility (n = 4, 1 RSD) of Pb content is 0.17–0.58%.

Among these reference materials, only Pb content (47.2 ± 1.7 μg/g) of JFCA-1 has been measured by atomic absorption spectrometry (AAS), as reported by Terashima et al. [26]. The Pb elemental content of JCFA-1 is 47.65 ± 0.07 μg/g in this study, showing an excellent agreement with Terashima et al. [26]. This study is the first to report the Pb elemental content for GBW08401, GBW11124, and GBW11125d. It is important to emphasize that our data recorded excellent reproducibility using the ID-TIMS method. Generally, among these CA and CFA reference materials, JCFA-1 and GBW11124 show better reproducibility (<0.20%, 1 RSD) of Pb elemental content and hence are more homogeneous.

### 3.2. Pb isotopic Ratio Results

As mentioned in Section 2.5, TIMS double spike analysis requires ^20x^Pb/^204^Pb ratios on two filament loads: a “natural” run with unspiked sample and a sample-spiked mixture run. The optimum mixture of sample and spike is the so-called “Q value”, which was calculated as ^204^Pb_sample_/^204^Pb_spike_ = 0.10–0.15 in this study, with a tolerance range of 0.03–0.65 [31,34] that was regarded as negligible uncertainty magnification. Both JB-3 and BCR-2 basalt standards were analyzed to verify the reliability of the sample digestion method, chemical procedure, and TIMS measurement. As shown in Table 5, the Pb isotopic ratios data for JB-3 and BCR-2 show good agreement with previously published data [29,30,32,33,36,37,38,45]. This demonstrates the whole analysis process as outlined in this paper is reliable.

**Figure 2:**
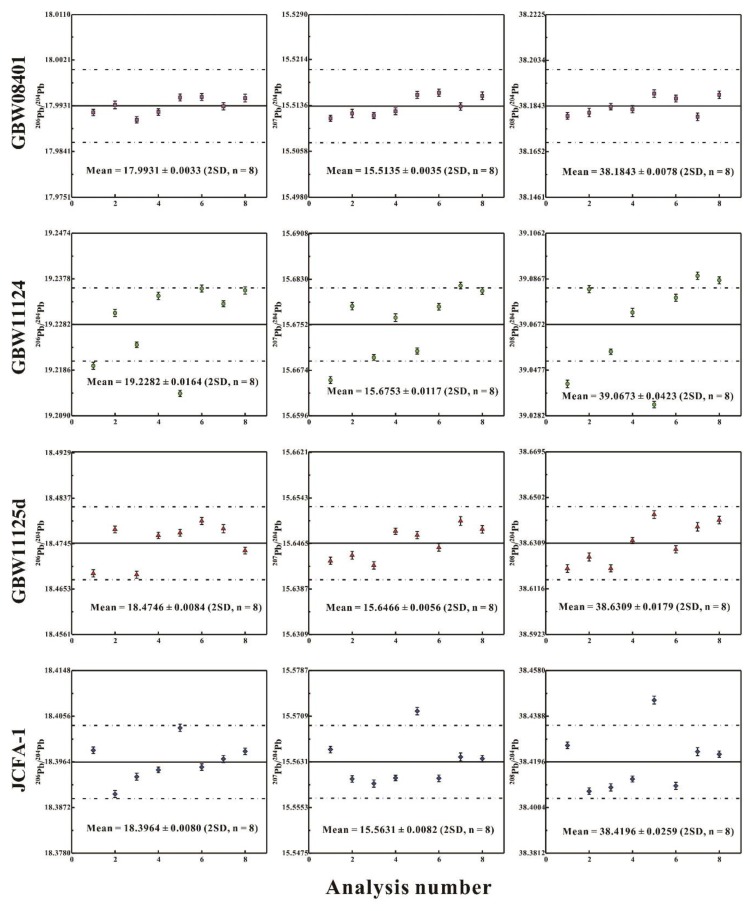
Pb isotope ratios in four CFA and CA reference materials. The dashed line area defines the error of ± 0.04% (2 RSD) of Pb isotope ratios.

Pb isotopic ratios measured by TIMS are listed in Table 5 and shown in Figure 2, along with their individual within-run precision (2 SE). It can be seen that good internal precisions were achieved in each run, which was between ± 0.0006 and ± 0.0008 for ^206^Pb/^204^Pb, between ± 0.0005 and ± 0.0008 for ^207^Pb/^204^Pb, between ±0.0012 and ± 0.0024 for ^208^Pb/^204^Pb, between ± 0.00001 and ± 0.00002 for ^206^Pb/^207^Pb, and between ± 0.00002 and ± 0.00004 for ^208^Pb/^206^Pb. During data collection, a long-life and stable Pb ion beam signal was obtained, typically with ^208^Pb of 4.5–6.5 V. This demonstrates that the purity of Pb fractions after chemical separation was appropriate for TIMS measurements.

This study is the first to report Pb isotopic ratios of reference materials (GBW11125d, GBW11124, and GBW08401), thus no previously published reference values are available for comparison. To obtain high reliable data of Pb isotope ratios, eight replicate analyses were determined for each of the reference materials (GBW08401, GBW11124, GBW11125d, and JCFA-1). As shown in Table 5 and Figure 2, generally, the external precision reproducibility (2 RSD, n = 8) of Pb isotopic ratios in these CRMs are good, except for GBW11124. GBW11124 shows poor reproducibility, which may be ascribed to either sample inhomogeneity or contamination during processing (crushing and pulverization), although we favor the former interpretation. In comparison to Sr and Nd isotope systems, Pb is more easily contaminated during the original preparation of the CRM samples [32]. As for GBW08401, GBW11125d, and JCFA-1, the external precision (2 RSD) reproducibility of ^206^Pb/^204^Pb, ^207^Pb/^204^Pb, ^208^Pb/^204^Pb, ^206^Pb/^207^Pb, and ^208^Pb/^206^Pb are narrowly variable i.e., 0.018–0.046%, 0.022–0.053%, 0.020–0.067%, 0.010–0.038%, and 0.016–0.045%, respectively. Generally, ^206^Pb/^207^Pb values among the five Pb isotopic ratios in these CRMs show the best reproducibility (<0.04%, 2 RSD), even achieving 0.010% for GBW08401.

Among these reference materials, only Pb isotope ratios of JFCA-1 have been measured by multi-collector–inductively coupled plasma–mass spectrometry (MC-ICP-MS) [36]. However, Hattori et al. [36] only conducted one analysis of JCFA-1 and no repeated measurements were undertaken. Hence, it is unknown whether the Pb isotope compositions of JCFA-1 are homogeneous. As shown in Table 5, our repeated measurement data (n = 8) for JCFA-1 demonstrate that it is homogeneous for Pb isotope ratios, being in good agreement with the data reported by Hattori et al. [36].

## 4. Conclusions

Evaluating the accuracy and precision of Pb elemental content and isotopic values of unknown CFA and CA samples requires the use of characterized CRMs with similar matrices being analyzed. In this study, high precision Pb isotope ratio determinations were made on four commercially available coal fly ash as well as coal ash reference materials. The external precision of Pb isotope ratios (except for GBW11124) is generally better than 0.05% (2 RSD, n = 8), varying from 0.010% to 0.067% (2 RSD, n = 8) for all Pb isotope ratios. The reproducibility of Pb contents is better than 0.60% (1 RSD, n = 4). These results indicate that JCFA-1, GBW08401, GBW11124, and GBW11125d can be used as reference materials for validating Pb isotope ratios and Pb elemental contents in coal fly ash and coal ash samples. Among these reference materials, JCFA-1 and GBW11124 show the best reproducibility (<0.20%, 1 RSD) for Pb elemental contents and GBW08401 shows the best reproducibility (<0.022%, 2 RSD) for Pb isotope ratios. We consider this dataset of Pb elemental contents and Pb isotopes, when combined with our previously published Rb-Sr and Sm-Nd elemental content and Sr-Nd isotope data [22] for the same suite of CRMs, will provide fundamental support for tracing the emission pathway of CA and CFA in environmental science.

## Figures and Tables

**Table 1:** The compositions of used ^207^Pb spike and ^204^Pb^-207^Pb double spike (atom%).

Spikes	^204^Pb	^206^Pb	^207^Pb	^208^Pb
^207^Pb	0.1391	2.452	92.813	4.631
^204^Pb–^207^Pb	40.165	1.555	55.457	2.823

**Table 2:** Pb purification scheme using AG1-X8 resin.

Procedure	Eluting Reagent	Eluting Volume (mL)
Cleaning column	6.0 M HCl	4.0
Cleaning column	Milli-Q Water	5.0
Cleaning column	0.7 M HBr	1.0
Loading sample	0.7 M HBr	0.5
Rinsing	0.7 M HBr	4.2
Eluting Pb	6.0 M HCl	1.2

**Table 3:** Configuration for Pb isotope analysis.

Element	C	H1	H2	H3
**Pb**	^204^Pb	^206^Pb	^207^Pb	^208^Pb

**Table 4:** Pb elemental contents in coal fly ash, coal ash and silicate CRMs.

CRMs	GBW08401	GBW11124	GBW1125d	JCFA-1	Reported	JB-3	Reported	BCR-2	Reported
	Pb (μg/g)	Pb (μg/g)	Pb (μg/g)	Pb (μg/g)	Value (μg/g)	Pb (μg/g)	Value (μg/g)	Pb (μg/g)	Value (μg/g)
	36.62	41.14	63.93	47.64	47.2 (Ref. 26)	5.33	5.58 (Ref. 41)	10.95	11.02 (Ref. 32)
	36.66	41.15	63.34	47.76		5.34	5.04 (Ref. 42)	10.98	10.50 (Ref. 43)
	36.93	41.01	63.88	47.59		5.29	5.035 (Ref. 45)	10.84	10.90 (Ref. 44)
	36.75	41.16	64.24	47.61		5.35		10.91	
Mean	36.74	41.12	63.85	47.65		5.33		10.92	
SD	0.14	0.07	0.37	0.08		0.03		0.06	
RSD (%)	0.38	0.17	0.58	0.17		0.56		0.55	

SD: Standard deviation, RSD: Relative Standard Deviation.

**Table 5:** Pb isotope ratios in coal fly ash, coal ash and silicate CRMs.

CRMs	^206^Pb/^204^Pb	2 SE	^207^Pb/^204^Pb	2 SE	^208^Pb/^204^Pb	2 SE	^206^Pb/^207^Pb	2 SE	^208^Pb/^206^Pb	2 SE
GBW08401	17.9918	0.0006	15.5114	0.0005	38.1801	0.0014	1.15991	0.00001	2.12209	0.00002
GBW08401	17.9933	0.0007	15.5122	0.0007	38.1816	0.0017	1.15994	0.00002	2.12199	0.00003
GBW08401	17.9903	0.0006	15.5119	0.0005	38.1840	0.0013	1.15978	0.00001	2.12247	0.00002
GBW08401	17.9919	0.0006	15.5126	0.0006	38.1829	0.0015	1.15982	0.00001	2.12223	0.00002
GBW08401	17.9947	0.0007	15.5154	0.0006	38.1895	0.0015	1.15980	0.00001	2.12226	0.00002
GBW08401	17.9948	0.0007	15.5158	0.0006	38.1874	0.0014	1.15978	0.00001	2.12213	0.00002
GBW08401	17.9930	0.0007	15.5134	0.0006	38.1798	0.0016	1.15983	0.00001	2.12193	0.00002
GBW08401	17.9946	0.0008	15.5152	0.0006	38.1890	0.0016	1.15980	0.00002	2.12225	0.00003
Mean	17.9931		15.5135		38.1843		1.15983		2.12217	
2SD	0.0033		0.0035		0.0078		0.00012		0.00035	
2RSD (%)	0.018		0.022		0.020		0.010		0.016	
GBW11124	19.2195	0.0007	15.6657	0.0006	39.0419	0.0016	1.22685	0.00001	2.03137	0.00002
GBW11124	19.2306	0.0007	15.6784	0.0006	39.0823	0.0016	1.22657	0.00001	2.03230	0.00002
GBW11124	19.2239	0.0006	15.6696	0.0005	39.0556	0.0012	1.22683	0.00001	2.03161	0.00003
GBW11124	19.2342	0.0008	15.6764	0.0007	39.0724	0.0018	1.22695	0.00002	2.03140	0.00003
GBW11124	19.2137	0.0007	15.6707	0.0005	39.0330	0.0014	1.22609	0.00001	2.03152	0.00002
GBW11124	19.2358	0.0007	15.6783	0.0006	39.0787	0.0015	1.22690	0.00001	2.03157	0.00002
GBW11124	19.2325	0.0006	15.6819	0.0006	39.0881	0.0015	1.22642	0.00001	2.03239	0.00003
GBW11124	19.2354	0.0008	15.6810	0.0006	39.0862	0.0015	1.22667	0.00001	2.03200	0.00002
Mean	19.2282		15.6753		39.0673		1.22666		2.03177	
2SD	0.0164		0.0117		0.0423		0.00059		0.00081	
2RSD (%)	0.086		0.075		0.108		0.048		0.040	
GBW11125d	18.4685	0.0007	15.6435	0.0006	38.6202	0.0016	1.18058	0.00001	2.09114	0.00002
GBW11125d	18.4774	0.0007	15.6445	0.0007	38.6250	0.0017	1.18108	0.00002	2.09040	0.00003
GBW11125d	18.4682	0.0007	15.6427	0.0006	38.6202	0.0015	1.18063	0.00001	2.09117	0.00002
GBW11125d	18.4762	0.0006	15.6486	0.0005	38.6320	0.0013	1.18069	0.00001	2.09091	0.00002
GBW11125d	18.4767	0.0007	15.6479	0.0006	38.6430	0.0016	1.18078	0.00001	2.09145	0.00003
GBW11125d	18.4791	0.0007	15.6458	0.0006	38.6283	0.0015	1.18109	0.00001	2.09038	0.00002
GBW11125d	18.4775	0.0008	15.6504	0.0008	38.6378	0.0018	1.18065	0.00001	2.09107	0.00003
GBW11125d	18.4731	0.0007	15.6489	0.0006	38.6407	0.0016	1.18047	0.00001	2.09173	0.00002
Mean	18.4746		15.6466		38.6309		1.18075		2.09103	
2SD	0.0084		0.0056		0.0179		0.00045		0.00094	
2RSD (%)	0.046		0.036		0.046		0.038		0.045	
JCFA-1	18.3987	0.0007	15.5652	0.0006	38.4265	0.0014	1.18204	0.00001	2.08854	0.00002
JCFA-1	18.3899	0.0007	15.5602	0.0006	38.4073	0.0014	1.18186	0.00001	2.08850	0.00002
JCFA-1	18.3934	0.0007	15.5594	0.0006	38.4089	0.0015	1.18214	0.00001	2.08819	0.00002
JCFA-1	18.3948	0.0006	15.5603	0.0005	38.4124	0.0013	1.18216	0.00001	2.08822	0.00002
JCFA-1	18.4032	0.0007	15.5718	0.0006	38.4456	0.0016	1.18183	0.00001	2.08907	0.00002
JCFA-1	18.3953	0.0007	15.5603	0.0006	38.4095	0.0015	1.18220	0.00001	2.08801	0.00002
JCFA-1	18.3970	0.0007	15.5640	0.0007	38.4238	0.0017	1.18203	0.00001	2.08860	0.00003
JCFA-1	18.3985	0.0006	15.5636	0.0006	38.4228	0.0013	1.18215	0.00001	2.08837	0.00002
Mean	18.3964		15.5631		38.4196		1.18205		2.08844	
2SD	0.0080		0.0082		0.0259		0.00028		0.00065	
2RSD (%)	0.043		0.053		0.067		0.024		0.031	
JCFA-1 ref value										
Ref. 36	18.399		15.565		38.426		1.18208		2.08842	
JB-3	18.2932	0.0007	15.5338	0.0006	38.2436	0.0015	1.17764	0.00001	2.09059	0.00002
JB-3	18.2936	0.0007	15.5357	0.0006	38.2479	0.0016	1.17752	0.00001	2.09079	0.00003
JB-3	18.2928	0.0008	15.5354	0.0008	38.2430	0.0020	1.17749	0.00002	2.09060	0.00003
JB-3	18.2936	0.0008	15.5365	0.0008	38.2480	0.0020	1.17746	0.00002	2.09078	0.00003
JB-3	18.2899	0.0007	15.5310	0.0007	38.2327	0.0017	1.17763	0.00001	2.09038	0.00003
JB-3	18.2923	0.0006	15.5349	0.0005	38.2413	0.0013	1.17750	0.00001	2.09056	0.00002
Mean	18.2926	0.0007	15.5346	0.0006	38.2427	0.0015	1.17754	0.00001	2.09062	0.00002
2SD	0.0028		0.0039		0.0112		0.00016		0.00031	
2RSD (%)	0.015		0.025		0.029		0.014		0.015	
JB-3 ref values										
Ref. 33	18.2942		15.5355		38.2496		1.17757		2.09080	
Ref. 37	18.2592		15.5356		38.2506		1.17531		2.09487	
Ref. 29	18.2900		15.5310		38.2320		1.17764		2.09032	
Ref. 30	18.2997		15.5228		38.2441		1.17889		2.08988	
Ref. 36	18.2910		15.5403		38.2500		1.17700		2.09119	
Ref. 45	18.2954		15.5380		38.2516		1.17746		2.09078	
Mean	18.2883		15.5339		38.2463		1.17731		2.09131	
BCR-2	18.7523	0.0006	15.6184	0.0005	38.7105	0.0012	1.20065	0.00001	2.06431	0.00003
BCR-2	18.7561	0.0008	15.6212	0.0009	38.7168	0.0024	1.20068	0.00002	2.06422	0.00004
BCR-2	18.7527	0.0007	15.6248	0.0008	38.7162	0.0023	1.20019	0.00002	2.06457	0.00003
BCR-2	18.7477	0.0008	15.6161	0.0006	38.7076	0.0015	1.20053	0.00001	2.06466	0.00002
BCR-2	18.7517	0.0007	15.6245	0.0006	38.7268	0.0015	1.20015	0.00001	2.06524	0.00002
BCR-2	18.7531	0.0006	15.6195	0.0005	38.7141	0.0013	1.20063	0.00001	2.06441	0.00002
Mean	18.7523		15.6207		38.7153		1.20047		2.06457	
2SD	0.0055		0.0069		0.0133		0.00048		0.00074	
2RSD (%)	0.029		0.044		0.034		0.040		0.036	
BCR-2 ref values										
Ref. 29	18.7520		15.6200		38.7150		1.20051		2.06458	
Ref. 30	18.7580		15.6240		38.7210		1.20059		2.06424	
Ref. 32	18.7529		15.6250		38.7237		1.20019		2.06494	
Ref. 38	18.7540		15.6230		38.7230		1.20041		2.06479	
Mean	18.7542		15.6230		38.7207		1.20042		2.06464	

SD: Standard deviation, RSD: Relative Standard Deviation.
